# Supplementary material for: A ballistic graphene superconducting microwave circuit
Source: Nat Commun. 2018 Oct 4;9:4069. doi: 10.1038/s41467-018-06595-2 (PMC6172216; doi:10.1038/s41467-018-06595-2)
Supplement: Supplementary file 1 — Supplementary Information [file 41467_2018_6595_MOESM1_ESM.pdf]

Supplementary Information for:

A ballistic graphene superconducting microwave circuit

Schmidt and Jenkins et al.

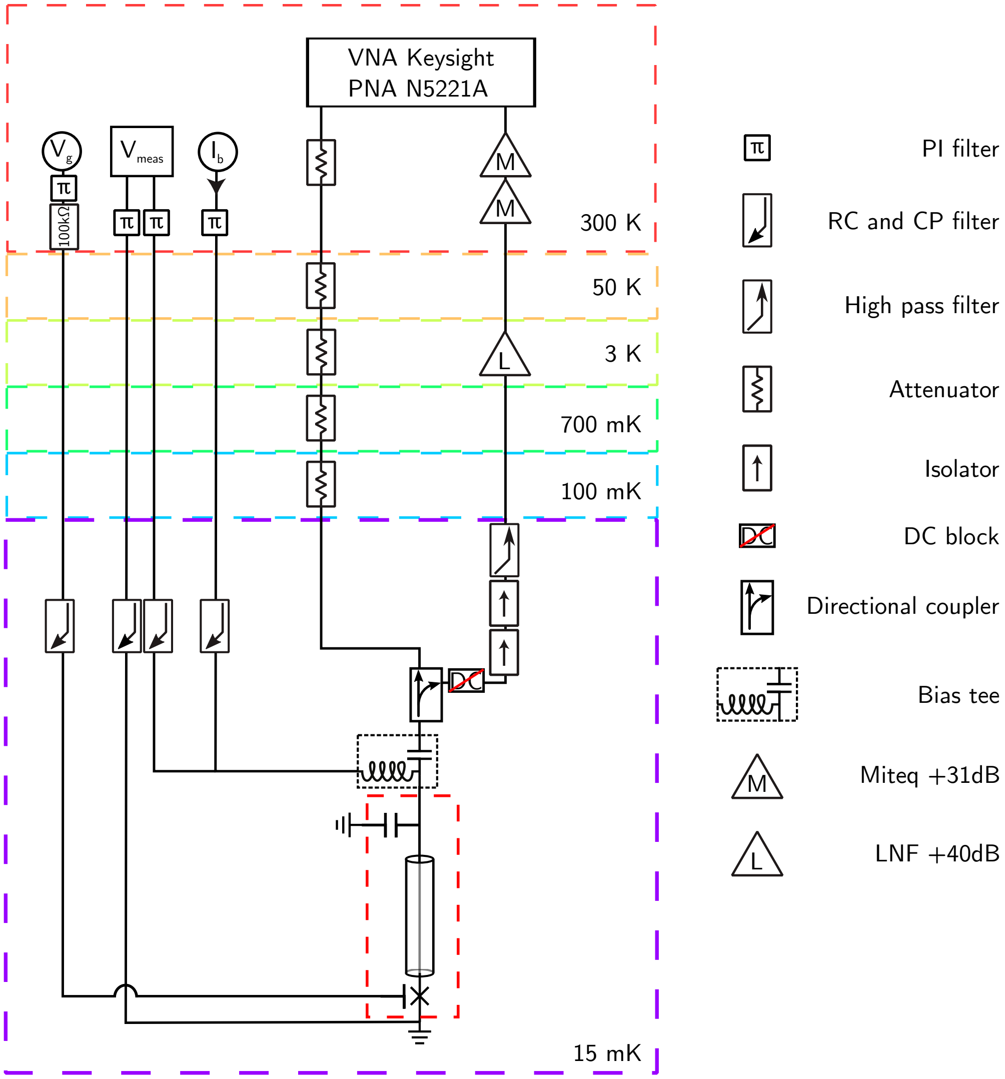

Supplementary Figure 1. **Sketched measurement setup.** Dashed red box at the bottom marks device outline.

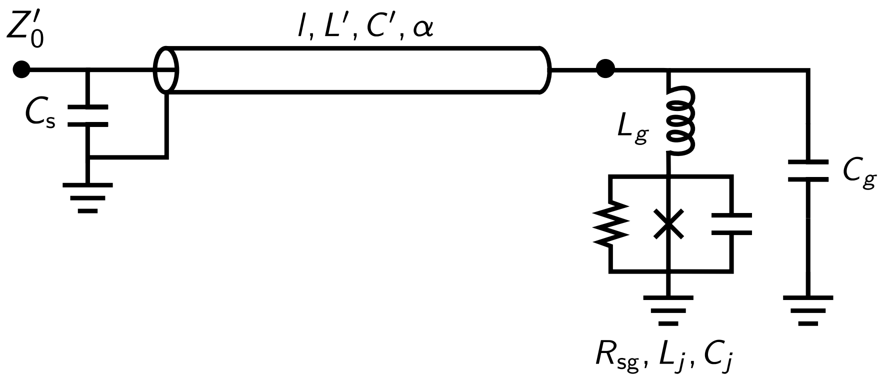

Supplementary Figure 2. **RF model for gJJ in cavity used for extraction of microwave parameters.** For the fitting procedure see Supplementary Note 2.

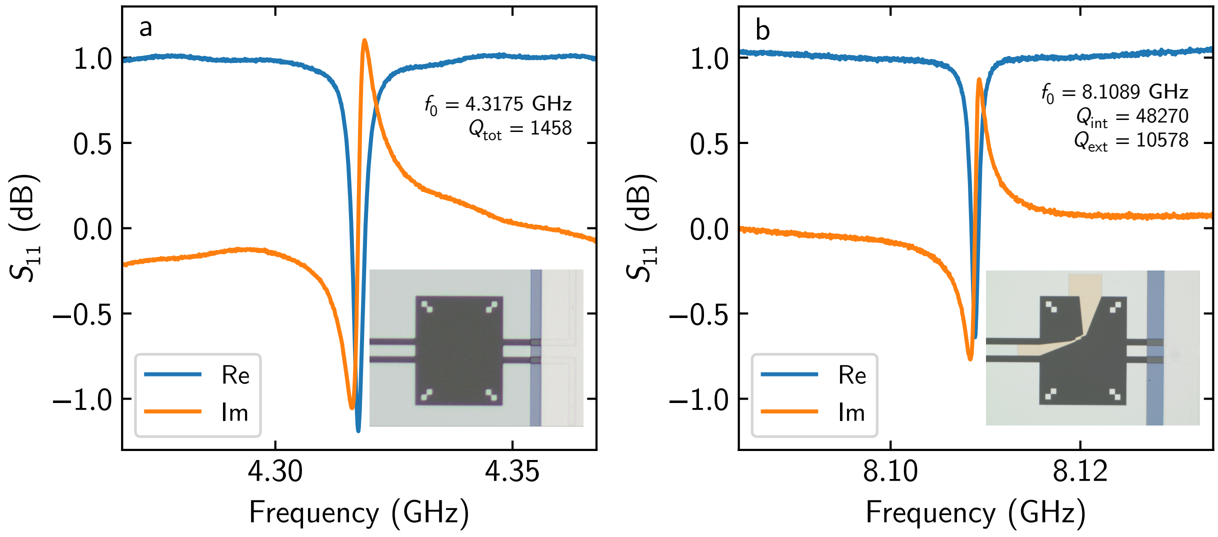

Supplementary Figure 3. **Reference samples for extraction of microwave parameters. a,** Open-ended cavity measurement of the real (imaginary) part of the reflection coefficient plotted in blue (orange). Inset: Optical micrograph of junction area of the measured device (open end). **b,** Shorted-cavity measurement with same lead geometry as the actual gJJ sample. Inset: Optical micrograph of junction area of the measured device (connected to ground).

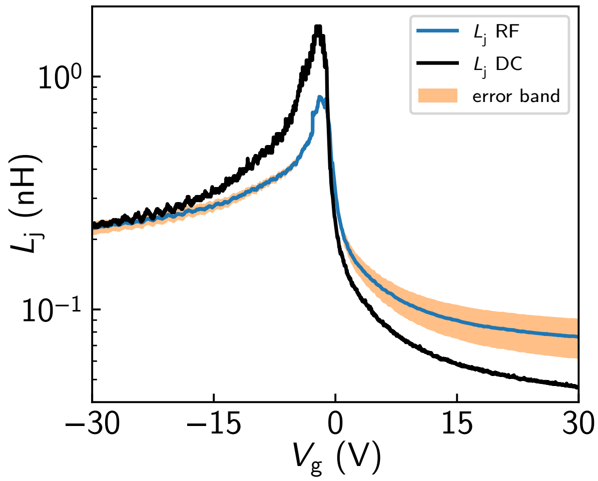

Supplementary Figure 4. **Josephson inductance extracted from RF and DC measurements, including error bands.** We plot here the same quantities as in Figure 3 of the main text but include error bands corresponding to minimum and maximum values originating from uncertainties in the circuit. The scales are identical to the plots in the main text.

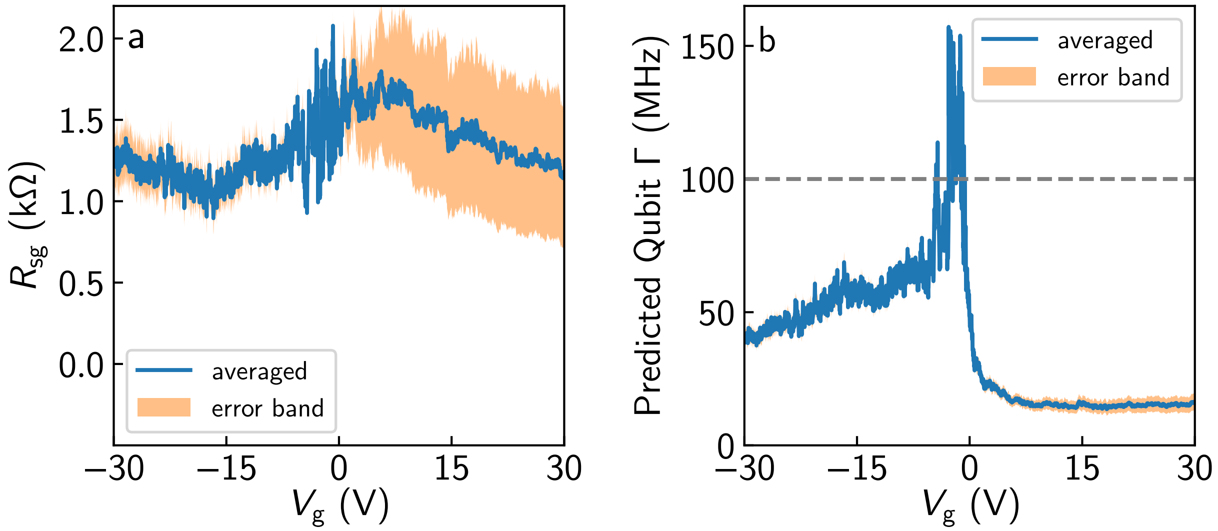

Supplementary Figure 5. **Subgap resistance from microwave cavity measurements, including error bands.** We plot here the same quantities as in Figure 4 of the main text, but include error bands corresponding to minimum and maximum values originating from uncertainties in the circuit. The scales are identical to the plots in the main text. **a,** Subgap-resistance including error band. **b,** Corresponding linewidth of the hypothetical transmon with error band.

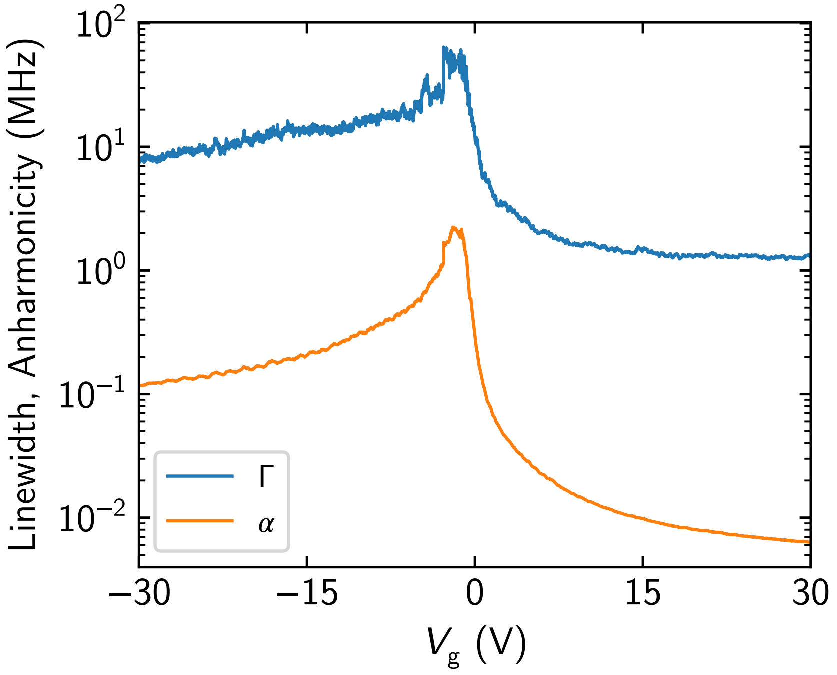

Supplementary Figure 6. **Anharmonicity and internal linewidth of current device, as described in Supplementary Note 3.** The calculated values of anharmonicity are always smaller than the measured linewidth meaning that this device cannot be considered a qubit in its current form.

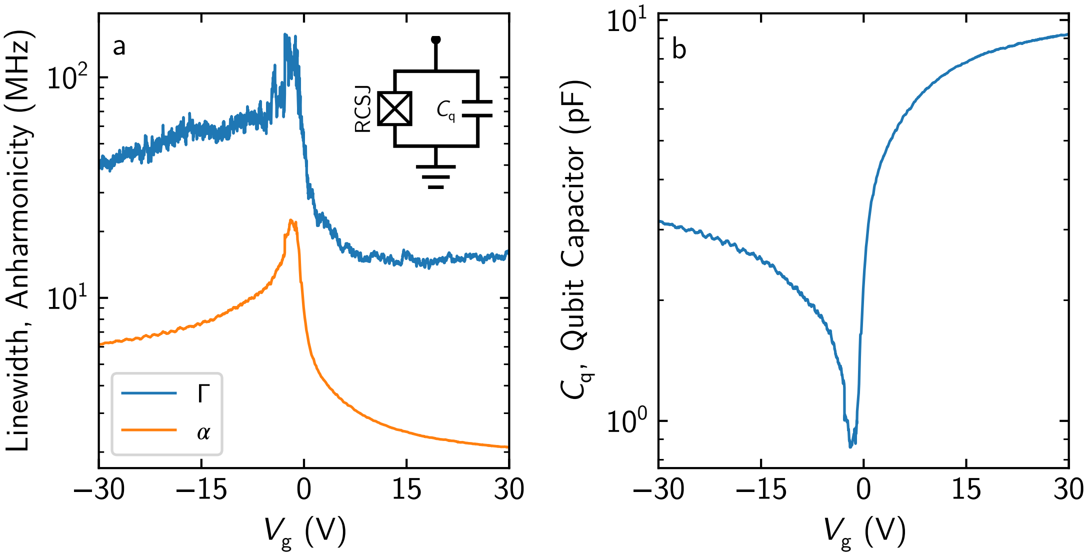

Supplementary Figure 7. **Anharmonicity and internal linewidth for design scenario A, as described in Supplementary Note 4. a,** We calculate the performance of the measured junction in a circuit shuch as the one shown in the inset. Setting the resonant frequency to $\omega_0 = 2\pi \cdot 6\,\mathrm{GHz}$, we then calculate the anharmonicity and linewidth of this hypothetical device. Also in this case we find that calculated values of anharmonicity are always smaller than the linewidth. **b,** Required value of capacitance C_q to maintain a resonant frequency of $\omega_0 = 2\pi \cdot 6\,\mathrm{GHz}$ as a function of V_g

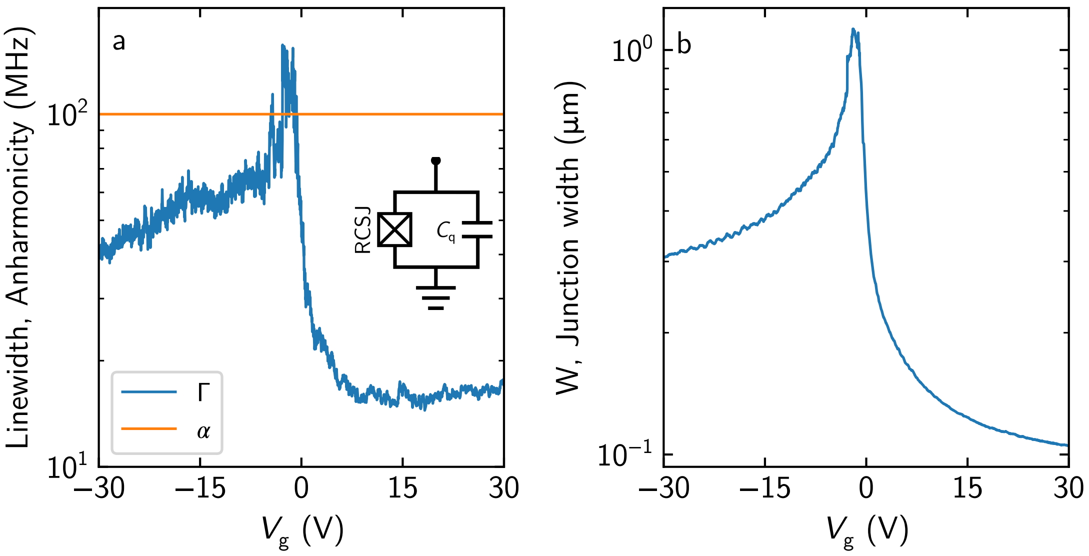

Supplementary Figure 8. **Anharmonicity and internal linewidth for design scenario B, as described in Supplementary Note 5. a,** We calculate the performance of a device whose capacitance and inductance are set by the requirement $\alpha = 100\,\text{MHz}$ and $\omega_0 = 2\pi \cdot 6\,\text{GHz}$. This means scaling the junction width as a function of V_g. The expected linewidth Γ is shown along with the designed anharmonicity. **b,** Required junction width to maintain a resonant frequency of $\omega_0 = 2\pi \cdot 6\,\text{GHz}$ and $\alpha = 100\,\text{MHz}$ as a function of V_g.

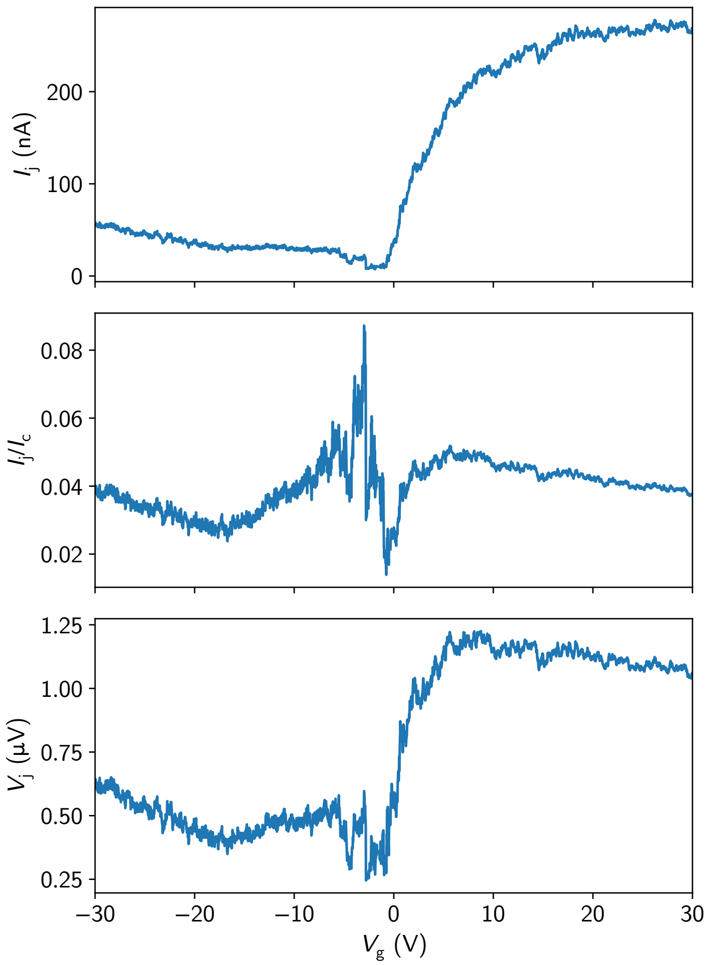

Supplementary Figure 9. **Current and voltage amplitude at junction for measurement in Figure 2c of main text.** The input power at the device is estimated to be approximately −122 dBm. Currents are well below the measured critical current of the junction, even near the charge neutrality point. The average voltage across the junction induced by the microwave tone is lower than 1 μV.

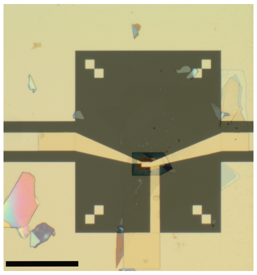

Supplementary Figure 10. **Microscope image of second graphene superconducting junction.** The flakes around the device are hBN residues from the transfer process. Scale bar 40 μm.

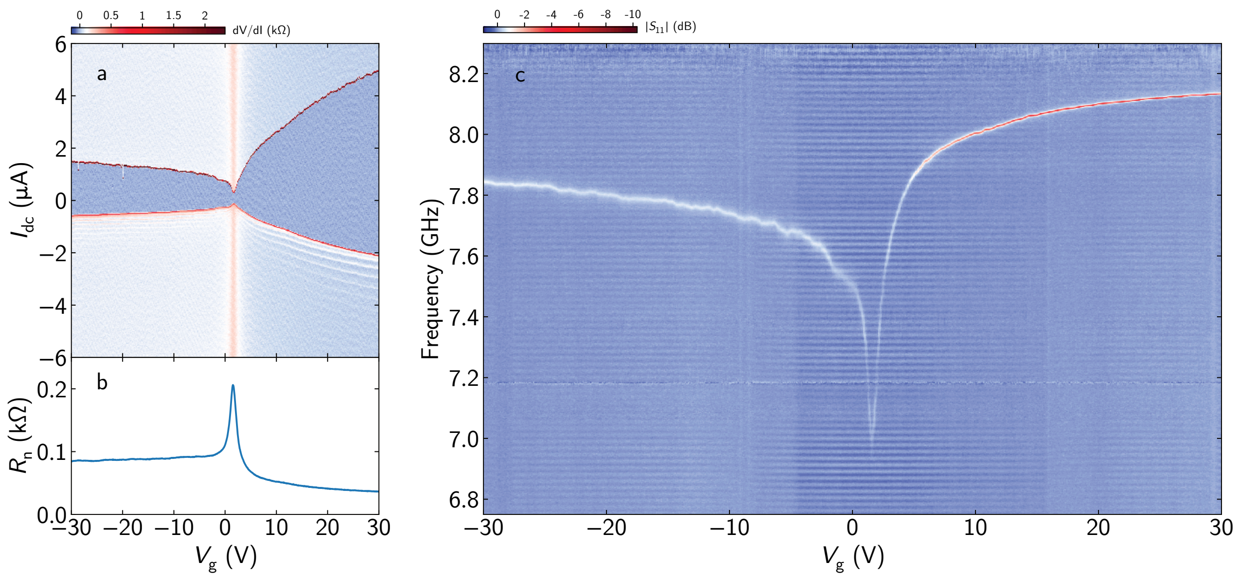

Supplementary Figure 11. **Observation of the Josephson inductance of a second graphene superconducting junction. a,** Differential resistance across the gJJ (Supplementary Figure 10) for a wide gate voltage range. Dark blue denotes area of zero resistance. **b,** Normal state resistance of the gJJ versus gate voltage. **c,** Microwave spectroscopy of the device in the superconducting state versus gate voltage, plotted as the amplitude of the reflection coefficient $|S_{11}|$ after background subtraction. Remarkably, its performance is broadly similar to the main text device (see Figure 2 of main text), despite having been stored at room temperature in a nitrogen box for ten months before measurement.

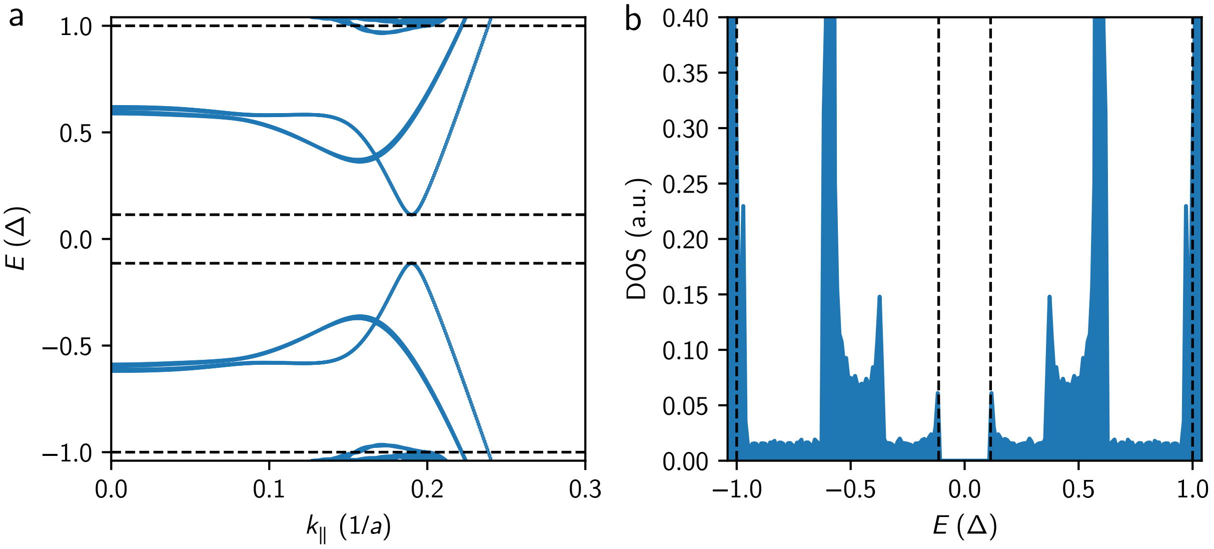

Supplementary Figure 12. **Dispersion and density of states of a gJJ, as described in Supplementary Note 7. a**, Simulated subgap dispersion for a graphene junction in the intermediate regime, $\Delta/E_{\mathrm{th}} = 1.542$ with $L_{\mathrm{N}} = 60$ and infinite lateral extension. Energy is scaled with respect to Δ, $k_{\|}$ in terms of momentum parallel to the SN-interface. **b**, By binning the energy dispersion we obtain the density of states as a function of energy. Various subgap peaks originating from ABS with high transverse momentum occur, while a hard gap remains, as indicated by the dashed horizontal lines.

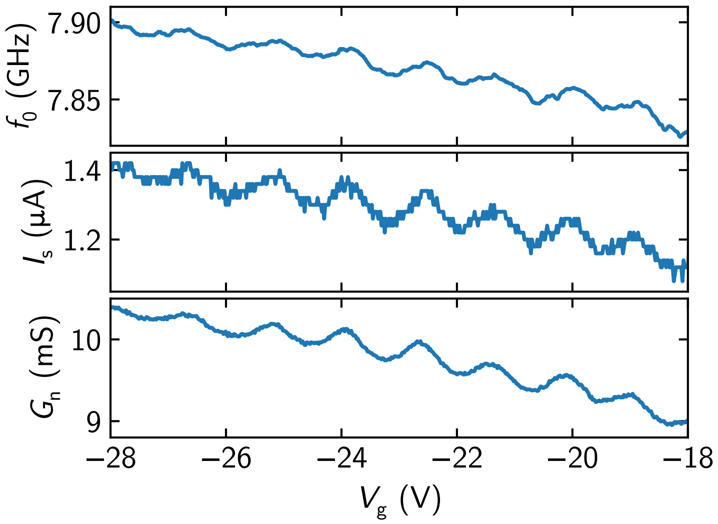

Supplementary Figure 13. **Correlating oscillations in DC and RF measurements.** We observe reproducible and matching oscillations in-phase oscillations of resonance frequency, critical current and normal state conductance in the npn-regime. We attribute these to interfering electron waves partially reflected from the SN interfaces at the graphene-superconductor contacts: Since NbTiN slightly n-dopes the contact region (hence the asymmetry in R_n as a function of gate voltage), pn-junctions form at the interface once the graphene is driven into the p-doped regime by the gate voltage. In the case of ballistic transport across the graphene sheet, the different charge carrier trajectories interfere with each other. Varying the gate voltage leads to a change in Fermi wavelength and hence an alternation of constructive and destructive interference, resulting in reduced and suppressed conductance, supercurrent, or inductance. This is akin to Fabry-Pérot oscillations of light waves in free space, bound by two mirrors. The observation of these Fabry-Pérot oscillations in graphene-based systems is uniformly taken as evidence of ballistic transport[1–13]. We therefore conclude that our device is also in the ballistic regime. We analyse these oscillations in Supplementary Figure 14.

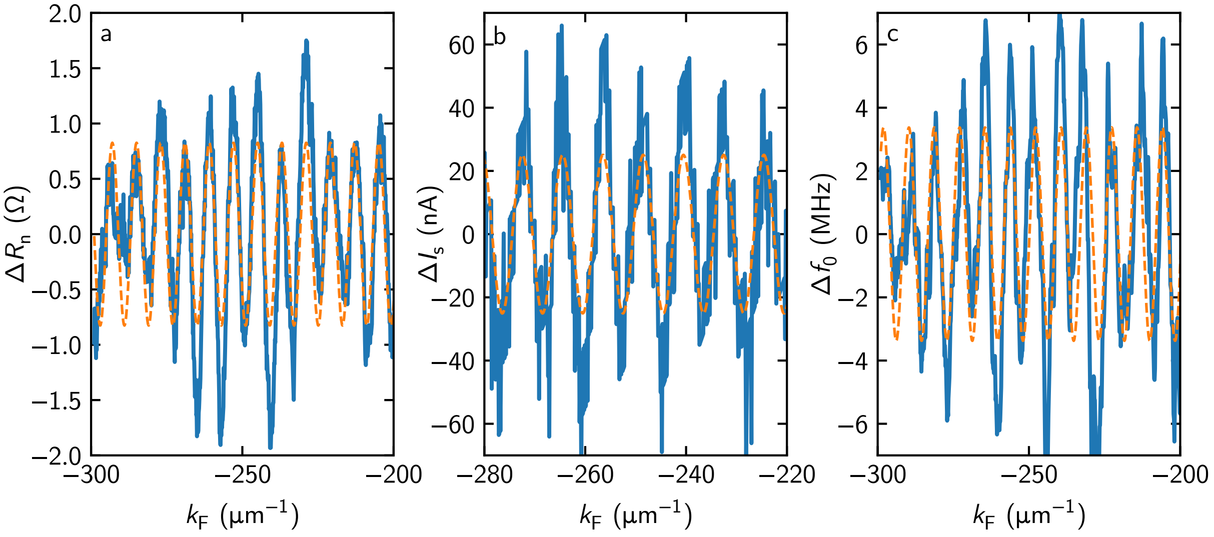

Supplementary Figure 14. **Fabry-Pérot oscillations in ballistic gJJ.** We observe FP oscillations in **(a)** $R_{\rm n}$, **(b)** $I_{\rm c}$ and **(c)** f_0. We can extract the length of the resonant cavity by fitting our oscillating signal with a sine, according to the resonance condition $2L_{\rm c} = m\lambda_{\rm F}, m \in \mathbb{N} \rightarrow 2L_{\rm c}k_{\rm F} = 2\pi m$. After subtracting a slowly varying background with a third-order polynomial[9], the fits for $R_{\rm n}$, $I_{\rm c}$ and f_0 (orange lines) independently yield $L_{\rm c} \approx 390\,\text{nm}$. This suggests a contact interface barrier of no more than 55 nm on each side. We can thus take $L_{\rm c}$ as a lower bound for the free momentum scattering and the phase coherence lengths, i.e. $l_{\rm mfp}, \xi > L_{\rm c}$.

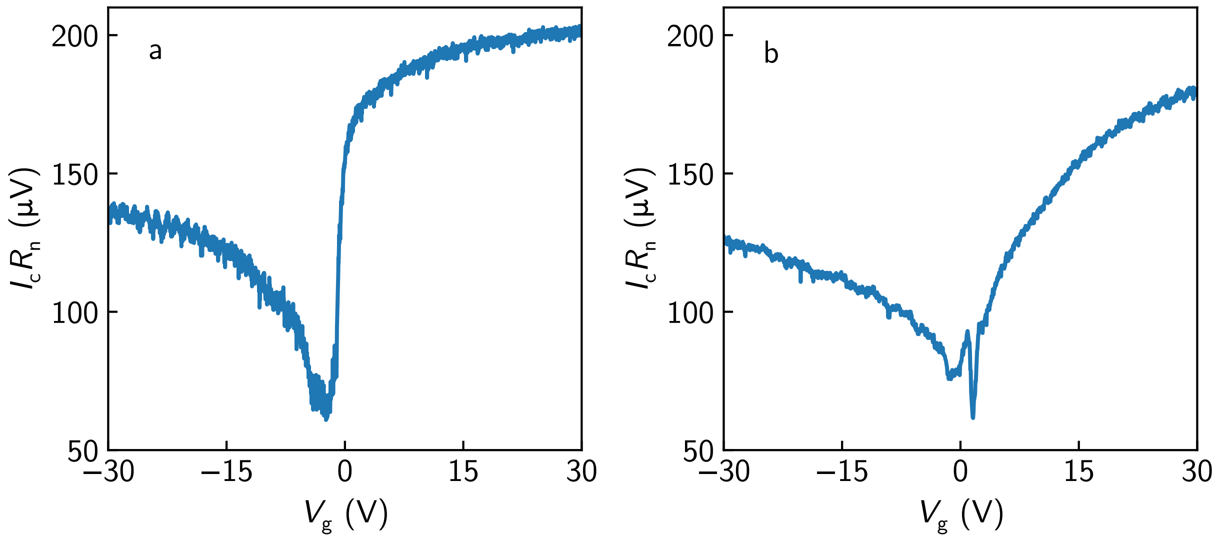

Supplementary Figure 15. I_cR_n **product of gJJ devices.** The I_cR_n product in Josepshon junctions is directly proportional to the gap voltage[14], with $I_cR_n \geq 2.08\Delta/e$ in the case of ballistic graphene junctions[15,16]. **a,** In our main device, this quantity saturates at approximately 200 µV for high n-doping, drops to 50 µV around CNP, and reaches up to 130 µV for high p-doping. We take the small dependence on gate voltage in high doping regime as further indication of ballistic transport[13,17]. Taking the bulk gap of the leads to be $\Delta = 1.764k_BT_c = 2\,\text{meV}$, our maximum $I_cR_n = 0.1\Delta$ which is much lower than the theoretically expected value. We attribute this to reduced contact transparency and our junction being in the long regime, where the Thouless energy $E_{th} = hv_F/L < \Delta$ is the dominant energy scale, limiting I_cR_n[18]. Our observation matches that of various other groups[8,11,13,17]. **b,** In contrast, the additional device lacks the saturating behaviour, and exhibits a lower I_cR_n product. This, in addition to the absence of FP oscillations, leads us to conclude that the latter device is non-ballistic, possibly due to a slightly longer normal region, or residual dirt (such as bubbles) in the graphene channel.

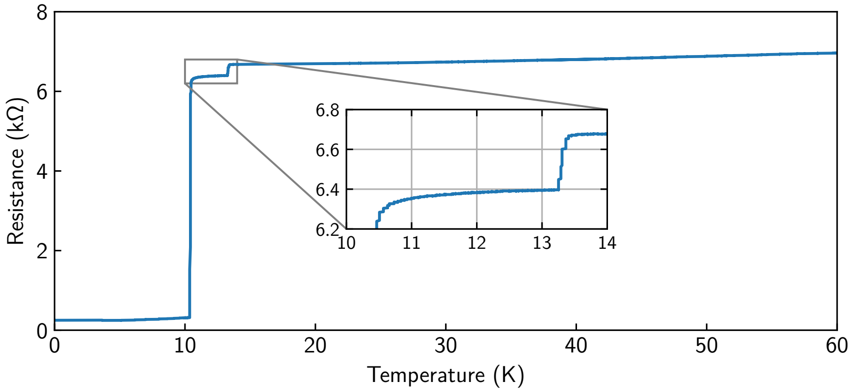

Supplementary Figure 16. **Critical temperature of MoRe and NbTiN.** Resistance versus temperature of the gJJ sample, measured during the initial cooldown, for a current bias of 1 µA without any gate voltage applied. The two jumps at 10.5 K and 13.2 K correspond to the critical temperature of MoRe and NbTiN, respectively. Below $T_{c,\mathrm{MoRe}}$, we measure a residual resistance of 250 Ω, which corresponds to the graphene sheet resistance for $V_g = 0\,\mathrm{V}$.

l (TL length)	6119 µm
C' (Capacitance per unit length)	0.148 48 nF/m
L' (Total inductance per unit length)	0.619 838 µH/m
C_s (Shunt coupler capacitance)	$\sim$ 27 pF
Z_0 (TL Characteristic impedance)	64.611 Ω
Z'_0 (Reference impedance)	50 Ω
v_{ph} (Phase velocity in TL)	$1.042\,38 \times 10^8$ m/s = 0.3477 c
$L'_g = \frac{\mu_0}{4}\frac{K(k_0'^2)}{K(k_0^2)}$ (Geometric inductance per unit length)	0.4277 µH/m
L'_k (Kinetic inductance per unit length)	0.1922 µH/m
L'_k/L' (Kinetic inductance fraction)	0.31
L_g (Geometric inductance of junction leads)	70 pH–100 pH
C_g (Geometric capacitance of junction leads)	4.7 fF
C_j (gJJ capacitance)	2 fF
α (Attenuation at 8.1089 GHz)	0.006 073 m^{-1}

Supplementary Table I. **Transmission line, coupler and junction parameters with kinetic inductance correction included, as described in Supplementary Note 2.**

Supplementary Note 1. FITTING ROUTINE FOR EXTRACTING THE RESONANCE FREQUENCY

The microwave response function of a capacitively shunted resonator in reflection geometry is given by[19]

$$\Gamma(\omega) = \frac{\kappa_{\rm ext} - \kappa_{\rm int} - 2i\Delta\omega}{\kappa_{\rm ext} + \kappa_{\rm int} + 2i\Delta\omega}, \tag{1}$$

where $\kappa_{\rm ext,int} = \omega_0/Q_{\rm ext,int}$ are the internal and external loss rates and $Q_{\rm ext,int}$ are the respective quality factors. $\Delta\omega = \omega - \omega_0$ is the frequency detuning from the resonance frequency ω_0.

The measured reflection coefficient must also include the effect of the connecting wires and devices between the network analyser and the device under test. The reflection coefficient is accordingly modified to incorporate this background:

$$S_{11} = B(\omega)\left(-1 + \frac{2\kappa_{\rm ext}e^{i\theta}}{\kappa_{\rm ext} + \kappa_{\rm int} + 2i\Delta\omega}\right) \tag{2}$$

The complex background $B(\omega)$ has the form:

$$B(\omega) = (a + b\omega + c\omega^2)e^{i(a'+b'\omega)}, \tag{3}$$

where a, b, c, a', b' are real parameters. We use this function to fit the measurement data and extract ω_0 and $\kappa_{\rm ext,int}$.

Supplementary Note 2. EXTRACTION OF PARAMETERS FROM MICROWAVE MEASUREMENTS

The schematic for the gJJ and cavity model can be seen in Supplementary Figure 2. A segment of a coplanar waveguide forms a cavity coupled on one side to an input line through a shunt capacitor. The far end of the transmission line (TL) segment has the gJJ modelled using an RCSJ model with an extra inductance and capacitance associated to the junction lead wires.

The parameters needed to characterize the system are described below, listed in Supplementary Table I and labelled in Supplementary Figure 2:

- The transmission line (TL) segment has a length l as well as a capacitance per unit length C' and inductance per unit length L'. TL losses are characterized by the

attenuation parameter α. It is worth noting that $L' = L'_\mathrm{g} + L'_\mathrm{k}$ includes a geometric contribution, L'_g, and kinetic inductance contribution[20], L'_k.

- The effective value of the shunt capacitance C_s. Since C_s parametrizes the external cavity coupling, this includes contributions from both the shunt capacitor and the external circuit. The different connectors, wires, and other microwave components introduce impedance mismatches and cable resonances in the input/output lines, changing the external coupling. We use C_s to reabsorb most of these effects, hence making it frequency dependent.

- The characteristic impedance of the input line Z'_0 taken as $50\,\Omega$, i.e., the VNA reference impedance.

- The gJJ is characterized by a junction inductance L_j, a junction capacitance C_j and subgap resistance R_sg.

- The junction leads also add a series inductance L_g and a shunt capacitance C_g.

With these inputs, the reflection response of the circuit can be calculated analytically and compared to the measured data. However, most of these parameters need to be calibrated and calculated first in order to deduce the junction parameters from the measurements. The different parameters and calibrations are set as follows:

- The cavity length is set by the design geometry of the cavity $l = 6119\,\mu\mathrm{m}$ and verified through microscope inspection.

- To determine the cavity L' and C' as well as the internal losses (related to α), several cavity measurements from the same batch as the final device were used. From fitting the fundamental mode resonances of these calibration samples we extracted values for L', C', α that we use for the final device. The samples used were:
  - A cavity with no junction at the end (Supplementary Figure 3a). This means that the fundamental mode frequency is approximately half that of the final device ($\lambda/4$ vs $\lambda/2$ boundary conditions). From this measurement and the physical geometry of the cavity, we deduce values for C', L'.

  - A cavity with a short at the end with the same shape as the final junction leads (Supplementary Figure 3b). This cavity was used to calibrate the loss parameter α associated to resistive and dielectric losses of the transmission line cavity. In principle, the losses are frequency dependent with higher losses at higher frequencies. Since this loss rate was obtained at the high end of the frequency range and is used for all our frequencies, the extracted loss rates are expected to overestimate the actual losses.

- The leads series inductance L_{g} and shunt capacitance C_{g} as well as the junction capacitance C_{j} were calculated using numerical simulation of the geometry (*COMSOL* v5.3 (COMSOL Inc., 2017) and *Sonnet* v16.54 (Sonnet Software Inc., 2017)). The contribution of the capacitances C_{j} and C_{g} are expected to be small compared to C_{s}. The impedances of these (parallel) capacitances are much larger than the typical impedances of the other circuit elements (L_{j} or R_{sg} for example).

- Additionally, L_{g} is swept between two extreme values given by our simulations representing a range of possible kinetic inductance values for NbTiN, the superconductor used in our leads. This gives the error band shown in Supplementary Figures 4 and 5.

With this, we are left with three free parameters: L_{j}, R_{sg}, C_{s}. These are determined from fitting the model to the microwave response of the final device as a function of applied gate voltage V_{g}. In broad terms, L_{j} sets the device resonance frequency, R_{sg} sets the internal quality factor (or loss rate) while C_{s} sets the external quality factor (or coupling). We note also that points around $V_{\mathrm{g}} = V_{\mathrm{CNP}}$ fall into a very undercoupled cavity regime, making the resonance peak visibility very low in some cases. This results in some of our fits not converging to the measured curve and producing absurd results. Since some of these peaks are not clearly fittable given the measured background, we have opted to reject these few low visibility traces from the final fitted parameter plots.

Supplementary Note 3. FEASIBILITY OF A GRAPHENE JJ TRANSMON QUBIT

In this section we provide an additional discussion on the feasibility of a graphene based transmon qubit.

We first consider the device as presented in the main text. To calculate the anharmonicity of this device we use techniques from the black box quantization method[21]. According to this method, the value of the anharmonicity α is then given by

$$\alpha = \frac{2e^2}{L_\mathrm{j}\omega_0^2(\mathrm{Im}(Y'(\omega_0)))}, \tag{4}$$

where L_j is the Josephson inductance of the junction, ω_0 is the resonant frequency of the circuit, Y is the admittance of the circuit seen from the junction terminals (including its own admittance) and Y' its derivative with respect to frequency. The resonance frequency ω_0 then corresponds to the condition $\mathrm{Im}Y(\omega_0) = 0$ and the derivative at this point $Y'(\omega_0)$ can be computed.

As can be seen in Supplementary Figure 6, the calculated anharmonicity for our main device is always smaller than the measured linewidth. Therefore it does not qualify as a qubit in its current state.

Supplementary Note 4. DESIGN SCENARIO A – MEASURED GRAPHENE JUNCTION IN FIXED FREQUENCY TRANSMON

While our device is not immediately a qubit, some improvements are possible. Most notably, the junction inductance is diluted by the cavity inductance, resulting in a low participation ratio in the total circuit inductance. We can therefore pose the question of what would the performance of a transmon be that contained only our graphene Josephson junction as its inductive element. This circuit is shown in the inset in Supplementary Figure 7a and consists of the junction in parallel with a shunt capacitor C_q. The value of this capacitance is set by the requirement that the frequency of the transmon be $\omega_0 = 2\pi \cdot 6\,\mathrm{GHz}$. Given the measured values of L_j as a function of applied gate voltage, we can then obtain the anharmonicity as:

$$\alpha = \frac{e^2}{2C_\mathrm{q}}. \tag{5}$$

The result is shown in Supplementary Figure 7a along with the projected linewidth of the device $\Gamma = (R_\mathrm{sg}C_\mathrm{q})^{-1}$. Although the situation is improved in this case, the anharmonicity is still substantially lower than the calculated linewidth. This is due to the fact that we are using a rather wide junction with a somewhat high critical current value and, therefore, a low inductance value. To keep the frequency at the chosen $\omega_0 = 2\pi \cdot 6\,\mathrm{GHz}$, the necessary

capacitance is then too large to make a qubit. This could be resolved by making our junction narrower, hence increasing its inductance, as we shall see below.

Supplementary Note 5. DESIGN SCENARIO B – ADJUSTED WIDTH GRAPHENE JUNCTION IN FIXED FREQUENCY AND ANHARMONICITY TRANSMON

In this case we consider the same circuit as in the previous case. Now, however, we fix the capacitance so that the anharmonicity $\alpha = 100\,\mathrm{MHz}$. This sets the value of our capacitance $C_\mathrm{q} \simeq 0.2\,\mathrm{pF}$. Since we also keep the requirement that $\omega_0 = 2\pi \cdot 6\,\mathrm{GHz}$, our junction inductance is fixed to a value of $L_\mathrm{j} = (\omega_0^2 C_\mathrm{q})^{-1} \simeq 3.5\,\mathrm{nH}$. Given these requirements and the measured values of inductance for our device, we can deduce what junction width would be necessary at each gate voltage V_g to produce the required inductance.

Here we make the assumption that both L_j and R_sg scale with the inverse of the junction width, i.e., approximately as $\propto W^{-1}$. This should be the case for L_j since $L_\mathrm{j} \propto= I_\mathrm{c}^{-1} \propto R_\mathrm{n} \propto W^{-1}$ since the $I_\mathrm{c} R_\mathrm{n}$ product in a ballistic junction is constant[15]. R_sg does not necessarily have to scale as R_n. It does, however, depend on the number of conduction channels available and on the graphene proximity gap. The number of channels should scale linearly with the width of the junction while the proximity gap should increase as high transverse momentum channels are suppressed. It is therefore reasonable to assume that R_sg scales at least as fast as L_j.

With these assumptions we can then calculate the required width and expected linewidth shown in Supplementary Figure 8. In this case there is an ample range of gate voltages that comply with the condition $\Gamma < \alpha$. The required junction widths are always above 100 nm, a limit that is within reach of state of the art fabrication techniques. It is on this basis that we propose that it is feasible to construct a graphene based transmon qubit.

Supplementary Note 6. HYSTERESIS OF THE JUNCTION SWITCHING CURRENT

The observed hysteresis in the switching current of our devices (see Figure 2a of main text, and Supplementary Figure 11a) could have various origins. A valid estimation of the relevant

Stewart-McCumber parameter[14], $\beta_{\mathrm{C}} = 2\pi I_{\mathrm{c}} R^2 C/\Phi_0$, is not straightforward because there is always the question of how much capacitance of the leads going to the junction should be included. In principle, for example in DC measurements, even a portion of the wires going up the cryostat could be arguably relevant, up to a point where the inductance of these wires "chokes" the capacitance contribution.

We here discuss several estimates of possible relevant capacitances that could enter into β_{C}, where we assume a typical $R = 50\,\Omega$ and $I_{\mathrm{s}} = 5\,\mu\mathrm{A}$. First, we note that the "geometric" capacitance of a parallel plate capacitor formed between the superconducting leads across the BN/G/BN stack yields a negligible value on the order of a few tens of atto Farads. More important is the "local" stray capacitance of the junction which we have simulated in *COMSOL* v5.3 (COMSOL Inc., 2017) and *Sonnet* v16.54 (Sonnet Software Inc., 2017). If we include the leads up to a distance of $5\,\mu$m from the junction, the relevant $C = 2\,\mathrm{fF}$ and $\beta_{\mathrm{C}} = 0.08$. We also simulated the capacitance of the leads that go from the junction to the surrounding ground plane and to the CPW cavity, giving $C = 6.7\,\mathrm{fF}$ and $\beta_{\mathrm{C}} = 0.25$. Of course, there is also likely a relevant capacitance contribution from the center conductor of the CPW to ground. For this, we can make a rough estimate of the total CPW center conductor capacitance of 909 fF and a resulting $\beta_{\mathrm{C}} = 35$, reaching far into the underdamped regime. Finally, one could also include the shunt capacitor of 27 pF, which would give $\beta_{\mathrm{C}} > 1000$. The last two are likely not completely relevant, since at the Josephson frequency associatated with the finite bias state of the junction ($\omega_{\mathrm{P}} = \sqrt{2\pi I_{\mathrm{c}}/(\Phi_0 C)} = 24\,\mathrm{GHz}$), the shunt capacitor will not charge through the inductance of the center wire of the cavity. More likely, the relevant β_{C} includes some reasonable contribution of the CPW capacitance: for example, assuming $C = C_{\mathrm{CPW}}/10 = 90\,\mathrm{fF}$ would give a $\beta_{\mathrm{C}} = 3.4$ In addition to these damping effects, self-heating effects inside the SNS junction could further contribute to a hysteretic IVC[22,23].

Supplementary Note 7. SIMULATION OF SUB-GAP DENSITY OF STATES

To gain further insight into the underlying mechanisms of our junction, we model the density of states (DOS) of a gJJ similar to our device with the software package *Kwant* v1.3[24]. The relevant energies to consider are the bulk superconducting pairing potential Δ and

the effective round-trip time of the Cooper pairs inside the junction, the Thouless energy $E_{\mathrm{th}} = \hbar v_{\mathrm{F}}/L$. From the critical temperature of our NbTiN leads (see Supplementary Figure 16) we estimate[14] $\Delta = 1.764 k_{\mathrm{B}} T_{\mathrm{c}} \approx 2\,\mathrm{meV}$. Our device is then placed in the intermediate to long regime, $\Delta/E_{\mathrm{th}} \approx 1.52 > 1$.

The modelled system consists of a discretized 2D honeycomb lattice with infinite boundary conditions in y-direction. The superconducting areas are implemented by setting the pairing potential of these regions to a finite value, effectively making the graphene itself superconducting. For the simulation shown we assume full SN coupling, corresponding to a contact transparency $Tr = 1$. The simulated system size was $L_{\mathrm{N}} = 60$ and $L_{\mathrm{SC}} = 300$ (both in units of the graphene lattice constant $a = 0.214\,\mathrm{nm}$), while we adjusted the pairing potential such that the junction is in the intermediate regime, i.e. $L_{\mathrm{N}}/\xi = \Delta/E_{\mathrm{th}} = 1.52$. The dispersion is obtained by solving the eigenvalue problem of the Hamiltonian discretized onto the implemented system and plotting the energy values as a function of transverse momentum $k_{||}$ (see Supplementary Figure 12).

As expected, there are several Andreev Bound States (ABS) hosted below the bulk gap, significantly reducing $\Delta_{\mathrm{ind}} < \Delta_{\mathrm{bulk}}$ and opening possible dissipation channels for RF excitations. As the chemical potential $\mu \gg \Delta$, the subgap states do not change much with doping, in agreement with the relatively flat R_{sg} in Figure 4b of the main text. In two-dimensional JJs, the aspect ratio can also play a non-negligible role, as there can be a second effective Thouless energy related to the transverse length, or width of the junction, $E_{\mathrm{th}}^{||} = \hbar v_{\mathrm{F}}/W_{\mathrm{N}}$. Hence, as the aspect ratio increases, the DOS below the bulk gap can rise significantly. Alternatively, one can understand this via the subgap dispersion: ABS with lowest energies are those exhibiting large transverse momentum because their effective path length is longer. The wider the junction, the longer the maximum direct paths across it become, thus the increase in subgap DOS. With $W_{\mathrm{N}}/L_{\mathrm{N}} \approx 10$, this is a contributing factor in our device.

Note that this discussion gets more complicated when considering the contact interfaces between the normal and superconducting parts, as for reduced contact transparencies the subgap states are even further pushed towards zero energy.

We confirm the validity of our simulation by calculating the energies of both infinite and finite systems for various scaling factors. The infinite system is the limit of the finite system with aspect ratio $L_{\mathrm{N}} \ll W_{\mathrm{N}}$. For a very narrow gJJ (lateral extension comparable or equal to distance between superconducting contacts), the DOS is much lower below the bulk gap

compared to a very wide junction. The reason for this is the much higher level spacing for a narrow system that pushes additional states above the gap. Hence, to obtain a SNS system with hard and large induced gap, the normal part should be as narrow and short as possible.

We note that these peaks are not directly visible in our measurements, since instead of measuring the voltage drop across a current-biased JJ they require spectroscopy of the DOS via a tunnel probe, such as in Pillet *et al.* or Bretheau *et al.*[25,26].

SUPPLEMENTARY REFERENCES

[1] Liang, W. et al. Fabry - Perot interference in a nanotube electron waveguide. *Nature* **411**(6838), 665–669 (2001). doi:10.1038/35079517.

[2] Miao, F. et al. Phase-Coherent Transport in Graphene Quantum Billiards. *Science* **317**(5844), 1530–1533 (2007). doi:10.1126/science.1144359.

[3] Young, A. F. & Kim, P. Quantum interference and Klein tunnelling in graphene heterojunctions. *Nature Physics* **5**(3), 222–226 (2009). doi:10.1038/nphys1198.

[4] Cho, S. & Fuhrer, M. Massless and massive particle-in-a-box states in single- and bi-layer graphene. *Nano Research* **4**(4), 385–392 (2011). doi:10.1007/s12274-011-0093-1.

[5] Wu, Y. et al. Quantum Behavior of Graphene Transistors near the Scaling Limit. *Nano Letters* **12**(3), 1417–1423 (2012). doi:10.1021/nl204088b.

[6] Campos, L. et al. Quantum and classical confinement of resonant states in a trilayer graphene Fabry-Pérot interferometer. *Nature Communications* **3**(1) (2012). doi: 10.1038/ncomms2243.

[7] Rickhaus, P. et al. Ballistic interferences in suspended graphene. *Nature Communications* **4**, 2342 (2013). doi:10.1038/ncomms3342.

[8] Ben Shalom, M. et al. Quantum oscillations of the critical current and high-field superconducting proximity in ballistic graphene. *Nature Physics* **12**(4), 318–322 (2015). doi:10.1038/nphys3592.

[9] Calado, V. E. et al. Ballistic Josephson junctions in edge-contacted graphene. *Nature Nanotechnology* **10**(9), 761–764 (2015). doi:10.1038/nnano.2015.156.

[10] Amet, F. et al. Supercurrent in the quantum Hall regime. *Science* **352**(6288), 966–969 (2016). doi:10.1126/science.aad6203.

[11] Borzenets, I. V. et al. Ballistic Graphene Josephson Junctions from the Short

to the Long Junction Regimes. *Physical Review Letters* **117**(23) (2016). doi:10.1103/PhysRevLett.117.237002.

[12] Allen, M. T. et al. Observation of Electron Coherence and Fabry–Perot Standing Waves at a Graphene Edge. *Nano Letters* **17**(12), 7380–7386 (2017). doi:10.1021/acs.nanolett.7b03156.

[13] Zhu, M. et al. Supercurrent and multiple Andreev reflections in micrometer-long ballistic graphene Josephson junctions. *Nanoscale* **10**(6), 3020–3025 (2018). doi:10.1039/C7NR05904C.

[14] Tinkham, M. *Introduction to Superconductivity* (McGraw-Hill, Inc., New York), 2 edition (1996). ISBN 978-0-486-43503-9.

[15] Titov, M. & Beenakker, C. W. J. Josephson effect in ballistic graphene. *Physical Review B* **74**(4) (2006). doi:10.1103/PhysRevB.74.041401.

[16] Cuevas, J. C. & Yeyati, A. L. Subharmonic gap structure in short ballistic graphene junctions. *Physical Review B* **74**(18), 180501 (2006). doi:10.1103/PhysRevB.74.180501.

[17] Mizuno, N., Nielsen, B. & Du, X. Ballistic-like supercurrent in suspended graphene Josephson weak links. *Nature Communications* **4**, 2716 (2013). doi:10.1038/ncomms3716.

[18] Dubos, P. et al. Josephson critical current in a long mesoscopic S-N-S junction. *Physical Review B* **63**(6), 064502 (2001). doi:10.1103/PhysRevB.63.064502.

[19] Pozar, D. M. *Microwave Engineering* (Wiley, Hoboken, NJ), 4. ed edition (2012). ISBN 978-0-470-63155-3. OCLC: 785830420.

[20] Van Duzer, T. & Turner, C. W. *Principles of Superconductive Devices and Circuits* (Prentice Hall, Upper Saddle River, N.J), 2nd ed edition (1999). ISBN 978-0-13-262742-9.

[21] Nigg, S. E. et al. Black-box superconducting circuit quantization. *Phys. Rev. Lett.* **108**, 240502 (2012). doi:10.1103/PhysRevLett.108.240502.

[22] Courtois, H. et al. Origin of Hysteresis in a Proximity Josephson Junction. *Physical Review Letters* **101**(6) (2008). doi:10.1103/PhysRevLett.101.067002.

[23] Borzenets, I. V. et al. Phonon bottleneck in graphene-based Josephson junctions at millikelvin temperatures. *Physical Review Letters* **111**(2) (2013). doi:10.1103/PhysRevLett.111.027001.

[24] Groth, C. W. et al. Kwant: A software package for quantum transport. *New Journal of Physics* **16**(6), 063065 (2014). doi:10.1088/1367-2630/16/6/063065.

[25] Pillet, J.-D. et al. Andreev bound states in supercurrent-carrying carbon nanotubes re-

vealed. *Nature Physics* **6**(12), 965–969 (2010). doi:10.1038/nphys1811.

[26]Bretheau, L. et al. Tunnelling spectroscopy of Andreev states in graphene. *Nature Physics* **13**(8), 756 (2017). doi:10.1038/nphys4110.
